# Serum Albumin as a Marker of Microvascular Burden in Diabetic Retinopathy: Mediation by Diabetic Nephropathy in Uncontrolled Diabetes

**DOI:** 10.1155/joph/6568455

**Published:** 2026-09-16

**Authors:** Yabing Zhang, Jin Liu, Qiuli Yu, Xinjun Xue, Li Yuan

**Affiliations:** ^1^ Department of Ophthalmology, The Second Affiliated Hospital of Nanjing Medical University, Nanjing Jiangsu, 210000, China, njmu.edu.cn

**Keywords:** diabetic nephropathy, diabetic retinopathy, mediation analysis, NHANES, serum albumin

## Abstract

**Purpose:**

This study evaluated whether low serum albumin functions as an independent risk marker for diabetic retinopathy (DR) or primarily reflects renal microvascular damage, which frequently co‐occurs with DR. We also re‐examined the neutrophil‐to‐lymphocyte ratio (NLR) as a candidate inflammatory marker.

**Methods:**

Cross‐sectional analysis of 1107 adults with diabetes from the National Health and Nutrition Examination Survey (NHANES) 2005–2008. Three sequential logistic regression models were fitted: Model 1 (unadjusted), Model 2 (metabolic covariates), and Model 3 (Model 2 plus log‐transformed urinary albumin‐to‐creatinine ratio [UACR]). A mediation analysis with 2000 bootstrap resamples estimated the proportion of the albumin–DR association attributable to the UACR pathway in participants with HbA1c ≥ 7.0%. Sensitivity analyses applied the NHANES sampling weights, strata, and primary sampling units.

**Results:**

DR was present in 356 of 1107 participants (32.2%; 29.0% after weighting to the U.S. population). Each 1 g/dL increase in serum albumin was associated with a 39% reduction in the odds of DR in the unadjusted model (OR 0.61, 95% CI 0.41–0.89). The association attenuated after adjustment for metabolic and renal covariates (Model 3 OR 0.82, *p* = 0.399). In the HbA1c ≥ 7.0% subgroup (*N* = 502), Model 2 yielded an OR of 0.45 per 1 g/dL (95% CI 0.23–0.87, *p* = 0.019), which attenuated to 0.56 (*p* = 0.107) once UACR was added. Weighted models gave the same pattern (Model 2 OR 0.41, 95% CI 0.17–0.99, *p* = 0.047; Model 3 OR 0.44, *p* = 0.078). The indirect association through UACR was statistically significant (OR 0.79, 95% CI 0.61–0.92; bootstrap *p* < 0.001), giving a proportion mediated of 34.4%. NLR was not associated with DR in any adjusted model.

**Conclusions:**

In poorly controlled diabetes, low serum albumin behaved as a downstream marker of systemic microvascular leakage, with much of its association with DR mediated by concurrent renal damage. The design is cross‐sectional, so the temporal ordering assumed by mediation analysis cannot be verified and these estimates remain associational. Low albumin in this setting should still prompt assessment of the kidney and the retina at the same visit.

## 1. Introduction

Diabetic retinopathy (DR) is still among the most common preventable causes of blindness in working‐age adults. The global burden is rising as more people live long enough to face late complications [1, 2]. Chronic hyperglycemia, measured by HbA1c, remains the main driver of DR. However, it does not explain everything. Some well‐controlled patients still progress to sight‐threatening disease. Others with persistently poor control never do [3, 4]. Endothelial injury, low‐grade inflammation, and oxidative stress all contribute [5, 6]. Recent work has added detail here. Sustained hyperglycemia increases mitochondrial superoxide production and depletes antioxidant defenses in the retina, and a number of tissue and circulating markers of the resulting oxidative burden have now been characterized [7]. Low‐grade inflammation and early neuroretinal degeneration develop alongside the microvascular lesions that define clinical DR, and in some patients precede them [8]. Clinicians nevertheless still want inexpensive, widely available blood markers that reflect these processes, not just HbA1c.

Hyperglycemia‐driven endothelial dysfunction damages the blood–retinal barrier. This is seen as an early step in DR pathogenesis [9, 10]. Systemic inflammation advances the process by encouraging leukocyte adhesion and capillary occlusion. Oxidative stress adds to neurovascular injury. Two candidate markers have drawn attention. The neutrophil‐to‐lymphocyte ratio (NLR) is taken from a routine complete blood count, and several studies have linked it to DR severity. These findings often fade once confounders are properly adjusted for [11–13]. In a large prospective cohort, composite systemic inflammatory indices predicted ocular outcomes only modestly, and the strength of the association varied by disease [14]. The mechanistic literature suggests why. Mitochondrial stress responses and early neurodegeneration in the retina are local processes, and the biomarkers proposed for them are correspondingly tissue level [15]. Serum albumin, also measured in this blood panel, is even more common. It maintains oncotic pressure and serves as an antioxidant carrier [16, 17]. In diabetes, albumin levels fall when the glomerular filtration barrier leaks. Both glomerular and retinal barriers share much microvascular biology. Thus, a fall in serum albumin can signal leakage from both organs simultaneously [18, 19].

How a systemic marker should be interpreted also depends on disease stage. Permeability changes and capillary dropout dominate early DR. Advanced disease is a different matter: fibrovascular proliferation, vitreoretinal traction, and structural remodeling of the retinal layers that remain measurable after vitrectomy have removed the proliferative tissue [20]. The transcription‐factor networks driving this proliferative shift act within the retina and vitreous rather than responding to circulating substrate concentrations [21]. A blood marker of microvascular leakage may therefore indicate that the barrier is failing without indicating how far remodeling has gone.

This clinical observation brings a simple question. If low albumin in diabetes mainly signals renal microvascular leakage, its association with DR should mostly vanish once we account for kidney damage. We tested this idea in the NHANES 2005–2008 sample, using stepwise adjustment and a mediation analysis with bootstrap inference. To our knowledge, no previous study has quantified the mediating role of UACR in the albumin–DR association in a nationally representative sample.

## 2. Materials and Methods

### 2.1. Study Design and Population

Data came from two consecutive NHANES cycles, 2005–2006 and 2007–2008, conducted by the U.S. National Center for Health Statistics (NCHS) [22]. NHANES uses a stratified, multistage probability design to obtain a broadly representative sample of the noninstitutionalized U.S. population. The public‐use files release the sampling weights, strata, and primary sampling units needed to reproduce that design, and we use them in the weighted sensitivity analysis described in Section 2.5. The original protocol received ethics approval from the NCHS Research Ethics Review Board. Every participant provided written informed consent. Because we used the public‐use, deidentified dataset, no additional institutional review was required. Reporting follows the STROBE checklist for cross‐sectional studies (Supporting Table S1).

### 2.2. Inclusion and Exclusion Criteria

We classified a participant as having diabetes if any of three criteria were met: HbA1c ≥ 6.5%, a self‐reported physician diagnosis, or current use of insulin or an oral hypoglycemic agent. From this pool, we excluded participants without a gradable fundus photograph. We also excluded those missing key exposures (serum albumin, complete blood count, urinary albumin, or creatinine) and those missing required covariates (HbA1c, blood pressure, BMI, diabetes duration, C‐reactive protein, lipid profile). This left 1107 participants for analysis (Supporting Figure S1).

### 2.3. Outcome: DR

Both eyes were imaged with a Canon CR6‐45NM nonmydriatic camera, using two standard fields per eye (optic disc and macula). Graders at the University of Wisconsin–Madison reading center assigned severity scores with a modified Early Treatment Diabetic Retinopathy Study (ETDRS) scale. We dichotomized the outcome as a severity score of 14 or higher in either eye. This included microaneurysms, hemorrhages, hard or soft exudates, intraretinal microvascular abnormalities, or new vessels. Absence of all such lesions was classified as non‐DR.

### 2.4. Exposures and Covariates

Serum albumin was measured on the DxC800 system with the bromocresol‐purple dye‐binding method. NLR was the ratio of the absolute neutrophil count to the absolute lymphocyte count, both taken from the same routine complete blood count. UACR (mg/g) was calculated from spot urine albumin and creatinine.

Covariates were chosen a priori from variables known to influence DR risk: age (in years), sex, BMI (kg/m^2^, with obesity defined as BMI ≥ 30), HbA1c, diabetes duration (current age minus age at first diagnosis), systolic and diastolic blood pressures, hypertension, C‐reactive protein, LDL‐C, and triglycerides. Hypertension was defined from the measured readings as SBP ≥ 140 mmHg or DBP ≥ 90 mmHg, following JNC7. It is used only as a descriptive variable in Table 1 and as a stratifying variable in the subgroup analysis. It does not enter the adjustment set of any model, which uses systolic blood pressure as a continuous covariate instead. Supporting Table S2 reports what happens under the 2017 ACC/AHA threshold (≥ 130/80 mmHg). The variables entering Models 1–3 and the mediation analysis were handled by complete‐case selection, so none of them were missing in the final analytic sample; the exclusions this produced are shown in Supporting Figure S1. Two descriptive variables were merged in after the sample had been fixed and did contain missing values: waist circumference (41 participants, 3.7%) and diastolic blood pressure (83 participants, 7.5%). Both were imputed with the variable median for Table 1 only. LDL‐C and triglycerides were measured only in the morning fasting subsample and are reported as observed, without imputation. Exact counts are given in Supporting Table S3.

**TABLE 1:** Baseline characteristics of study participants stratified by DR status.

Characteristic	No DR (*n* = 751)	DR (*n* = 356)	*p*
Age (years), mean ± SD	62.9 ± 10.8	63.5 ± 10.7	0.359
Male sex, *n* (%)	364 (48.5%)	190 (53.4%)	0.144
Diabetes duration (years), median (IQR)	8.0 (3.0–8.0)	12.0 (8.0–19.2)	< 0.001
BMI (kg/m^2^), median (IQR)	31.4 (27.6–36.0)	30.4 (26.8–35.4)	0.038
Obesity (BMI ≥ 30 kg/m^2^), *n* (%)	468 (62.3%)	190 (53.4%)	0.006
Waist circumference (cm), mean ± SD	109.7 ± 15.2	107.5 ± 14.5	0.028
Systolic BP (mmHg), mean ± SD	133.2 ± 19.7	137.2 ± 22.2	0.003
Diastolic BP (mmHg), median (IQR)	70 (62–78)	70 (60–74)	< 0.001
Hypertension, *n* (%)	250 (33.3%)	138 (38.8%)	0.086
HbA1c (%), median (IQR)	6.7 (6.2–7.3)	7.5 (6.7–8.8)	< 0.001
Serum albumin (g/dL), mean ± SD	4.1 ± 0.3	4.1 ± 0.3	0.011
NLR, median (IQR)	2.0 (1.5–2.6)	2.0 (1.5–2.7)	0.111
CRP (mg/dL), median (IQR)	0.3 (0.1–0.8)	0.3 (0.1–0.6)	0.023
UACR (mg/g), median (IQR)	13.1 (6.5–33.1)	22.7 (10.2–94.9)	< 0.001
HDL‐C (mg/dL), mean ± SD	47.5 ± 13.8	49.4 ± 14.3	0.039
LDL‐C (mg/dL), mean ± SD^a^	105.4 ± 39.8	104.3 ± 38.3	0.783
Triglycerides (mg/dL), median (IQR)^a^	148.0 (103.5–215.0)	127.0 (95.2–190.0)	0.049

*Note:* Data are mean ± SD, median (IQR), or *n* (%). HbA1c, glycated hemoglobin; IQR, interquartile range.

Abbreviations: BMI, body‐mass index; BP, blood pressure; CRP, C‐reactive protein; DR, diabetic retinopathy; HDL‐C, high‐density‐lipoprotein cholesterol; LDL‐C, low‐density‐lipoprotein cholesterol; NLR, neutrophil‐to‐lymphocyte ratio; SD, standard deviation; UACR, urinary albumin‐to‐creatinine ratio.

^a^LDL‐C (observed *N* = 494) and triglycerides (observed *N* = 529) were measured only in the fasting morning subsample.

### 2.5. Statistical Analysis

Continuous variables are given as mean ± SD when approximately normal and as median (IQR) when skewed. Categorical variables are given as *n* (%). For between‐group comparisons, we used the *t*‐test for normally distributed continuous variables, the Mann–Whitney *U* test for non‐normally distributed ones, and the *χ* ^2^ test for categorical variables.

Three logistic regression models were fitted in sequence to estimate odds ratios (ORs) and 95% confidence intervals (CIs) for the association between serum albumin (or NLR) and DR. Model 1 was unadjusted. Model 2 added age, sex, BMI, systolic blood pressure, HbA1c, diabetes duration, and CRP. Model 3 added log (UACR + 1) on top of Model 2 to test whether the albumin signal survived adjustment for renal microvascular damage. In the HbA1c‐stratified subgroup analyses, HbA1c was not included as a covariate because it served as the stratifying variable. To examine the shape of the albumin–DR association, we fitted restricted cubic splines with knots at Harrell’s recommended percentiles. Three‐, four‐, and five‐knot specifications were compared on the Akaike and Bayesian information criteria, and departure from linearity was tested by a likelihood‐ratio test of the spline terms against the corresponding linear model. Both criteria favored three knots, placed at the 10th, 50th, and 90th percentiles (Supporting Table S4). Subgroup analyses were prespecified by age, sex, obesity, hypertension, and glycemic control. Each albumin × stratum interaction was tested with a Wald test on the multiplicative scale. Such tests have much less power than the tests of the corresponding main effects, so we also computed the effect size detectable at 80% power for the principal models and for the interaction test (Supporting Table S5).

Because the stepwise attenuation pattern suggested mediation by UACR, we conducted a mediation analysis in the HbA1c ≥ 7.0% subgroup. The mediator model was a linear regression of log (UACR + 1) on albumin and the same covariates as Model 2. The outcome model was a logistic regression of DR on albumin, log (UACR + 1), and those covariates. We applied the product‐of‐coefficients approach with a rare‐outcome logit approximation. We drew 95th percentile CIs and two‐sided *p* values from 2000 nonparametric bootstrap resamples (random seed = 20260527). The estimator carries the usual identification assumptions of mediation analysis, one of which is a temporal ordering running from exposure to mediator to outcome. That ordering cannot be verified in cross‐sectional data, so we report the decomposition as a quantitative description of the observed association structure and not as evidence of a causal sequence. The full variable and software specification are in Supporting Table S6.

The discriminative performance of Models 2 and 3 was compared using ROC curves and the area under the ROC curve. As a sensitivity check, we re‐ran the main models with albumin treated as quartiles (Supporting Table S7). The principal models were fitted without the NHANES survey weights because the mediation estimator we used is not implemented for survey‐weighted designs; weighting only part of the analysis would leave the regression and mediation results on a different footing. To establish whether that choice affects the findings, we refitted Models 1–3 as survey‐weighted logistic regressions using the 4‐year mobile examination center weight (WTMEC2YR/2) together with SDMVSTRA and SDMVPSU. Variances were obtained by Taylor‐series linearization, and the HbA1c strata were treated as design domains rather than as independent samples (Supporting Table S8). All analyses were performed in Python 3.9 using pandas, statsmodels, and scikit‐learn. A two‐sided *p* value below 0.05 was treated as statistically significant.

## 3. Results

### 3.1. Baseline Characteristics

Of the 1107 adults in the analysis, 356 (32.2%) had DR. Baseline characteristics by DR status are shown in Table 1. Participants with DR had longer‐standing diabetes and higher HbA1c (both *p* < 0.001). The mean serum albumin was 4.1 ± 0.3 g/dL in both groups, though the distributions differed modestly (*p* = 0.011). UACR was higher in the DR group (median 22.7 vs. 13.1 mg/g, *p* < 0.001), consistent with the renal–retinal axis. NLR did not differ between groups (median 2.0 in both, *p* = 0.111). Systolic blood pressure and the lipid profile were both modestly worse in the DR group. Weighted to the U.S. population, these 1107 participants correspond to a national DR prevalence of 29.0% among adults with diabetes, below the 32.2% observed in the unweighted sample.

### 3.2. Associations of Serum Albumin and NLR With DR

Table 2 lays out the stepwise results for serum albumin and NLR. Each 1 g/dL of albumin was associated with 39% lower odds of DR in the unadjusted model (OR 0.61, 95% CI 0.41–0.89, *p* = 0.011). The metabolic adjustment in Model 2 raised the OR to 0.65 and lowered the *p* value to 0.062. Adding log‐UACR in Model 3 removed the association entirely (OR 0.82, *p* = 0.399). NLR was not associated with DR in either model (Model 2: OR 1.06, *p* = 0.319; Model 3: OR 1.01, *p* = 0.885).

**TABLE 2:** Associations of serum albumin with DR in the full sample and HbA1c subgroups.

Population/subgroup	Model 1 OR (95% CI)	*p*	Model 2 (metabolic) OR (95% CI)	*p*	Model 3 (+UACR) OR (95% CI)	*p*
Full sample (*N* = 1107)	0.61 (0.41–0.89)	0.011	0.65 (0.41–1.02)	0.062	0.82 (0.51–1.31)	0.399
HbA1c < 7.0% (*N* = 605)	0.93 (0.50–1.70)	0.807	0.86 (0.45–1.67)	0.661	1.08 (0.55–2.10)	0.822
HbA1c ≥ 7.0% (*N* = 502)	0.61 (0.36–1.04)	0.068	0.45 (0.23–0.87)	0.019	0.56 (0.28–1.13)	0.107
P for interaction (Albumin × HbA1c)	—	—	0.167	—	—	—

*Note:* Odds ratios refer to a 1‐g/dL increase in serum albumin. Model 1: unadjusted. Model 2: adjusted for age, sex, BMI, systolic blood pressure, diabetes duration, and CRP, plus HbA1c in the full‐sample analysis. Model 3: Model 2 plus log (UACR + 1). UACR, urinary albumin‐to‐creatinine ratio.

The picture changed when we split the sample by glycemic control. In participants with HbA1c ≥ 7.0% (*N* = 502), the metabolic‐adjusted OR for albumin was 0.45 per 1 g/dL (95% CI 0.23–0.87, *p* = 0.019); once UACR entered the model, the estimate moved back to 0.56 and lost significance (*p* = 0.107). In participants with HbA1c < 7.0%, there was no signal in any model. The smaller subgroup thus reached significance where the larger full sample did not. This is not a contradiction. The estimate in the poorly controlled stratum lies about twice as far from the null as the full‐sample estimate (OR 0.45 vs. 0.65), which more than offsets the loss of sample size, and DR events are concentrated in that stratum (223 of 356). With those 223 events, the subgroup analysis could detect an OR of 0.38 or more extreme at 80% power. Its power against the estimate we observed was about 65%. For the full sample, the same figure was 46% (Supporting Table S5). The albumin × HbA1c interaction did not reach significance (*p* = 0.167), but that test was the least powerful of the three. Detecting the observed difference between strata at 80% power would have required a ratio of odds ratios of 0.29 or more extreme, against an observed ratio of 0.54, leaving the interaction test with about 28% power. These data do not settle the question either way. We treat the subgroup difference as a hypothesis for a larger sample, not as established effect modification.

Survey‐weighted estimates are reported in Supporting Table S8. Adding the sampling weights, strata, and primary sampling units left the pattern intact, with point estimates slightly further from the null. In the full sample, the albumin OR was 0.54 (95% CI 0.34–0.85, *p* = 0.010) unadjusted, 0.59 (0.35–1.00, *p* = 0.050) in Model 2, and 0.67 (0.38–1.19, *p* = 0.162) in Model 3. In the HbA1c ≥ 7.0% domain, it was 0.41 (0.17–0.99, *p* = 0.047) in Model 2 and 0.44 (0.18–1.10, *p* = 0.078) in Model 3. Weighted NLR estimates remained null (Model 2 OR 1.06, 95% CI 0.90–1.23, *p* = 0.478). The stepwise attenuation after UACR was added, which is the observation the mediation hypothesis rests on, and was therefore reproduced under the design‐based analysis. We report both sets of estimates rather than replacing one with the other since they answer different questions. The weighted models describe the U.S. adult diabetic population. The unweighted models describe the association within the analyzed sample, which is what the mediation decomposition operates on.

### 3.3. Dose–Response Relationship

**FIGURE 1:**
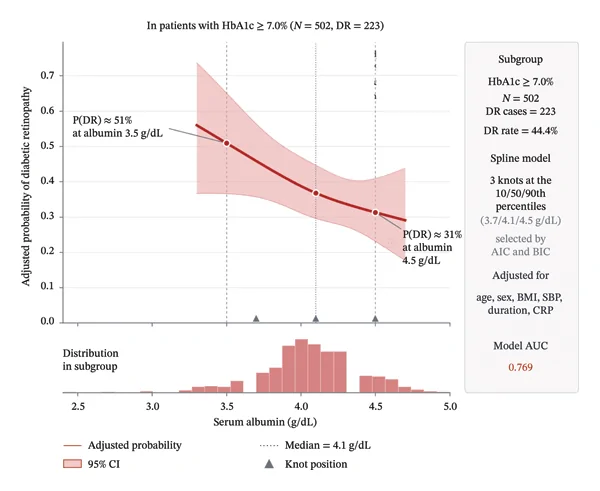
Restricted cubic spline of serum albumin and the predicted probability of diabetic retinopathy within participants with HbA1c ≥ 7.0%. The main panel shows the adjusted probability of diabetic retinopathy (DR) plotted against serum albumin in the poorly controlled subgroup (HbA1c ≥ 7.0%; *N* = 502, DR cases = 223). The curve was fitted using a multivariable logistic regression with a restricted cubic spline for albumin (3 knots at the 10th, 50th, and 90th percentiles, corresponding to 3.7, 4.1, and 4.5 g/dL), adjusted for age, sex, body‐mass index, systolic blood pressure, diabetes duration, and C‐reactive protein. The three‐knot specification was selected over four‐ and five‐knot alternatives by the Akaike and Bayesian information criteria (Supporting Table S4). The likelihood‐ratio test of the spline terms against a linear model was not significant (*p* = 0.664), so the curvature shown is descriptive rather than statistically established. The solid red line is the point estimate, and the shaded band is the 95% pointwise confidence interval. Triangles on the axis mark the knot positions. Three reference markers are plotted on the curve at albumin = 3.5 g/dL, the subgroup median (4.1 g/dL) and 4.5 g/dL. The lower strip shows the distribution of serum albumin in the subgroup. The spline model’s discriminative performance in this subgroup is AUC = 0.769. Continuous covariates were held at their subgroup medians for prediction. DR, diabetic retinopathy; HbA1c, glycated hemoglobin; AUC, area under the receiver‐operating‐characteristic curve.

Figure 1 shows the restricted cubic spline for albumin in the HbA1c ≥ 7.0% subgroup. Predicted DR probability falls steeply between albumin levels of about 3.0 and 4.2 g/dL and then flattens, giving the fitted curve a shallow L shape. After adjustment for age, sex, BMI, blood pressure, diabetes duration, and CRP, the predicted probability of DR is roughly 51% at albumin = 3.5 g/dL, 37% at the subgroup median of 4.1 g/dL and 31% at 4.5 g/dL. Both AIC and BIC preferred the three‐knot specification over four and five knots, but the likelihood‐ratio test of the spline terms against a linear model was not significant (*p* = 0.664; Supporting Table S4). The flattening at the upper end of the albumin range is therefore a feature of the fitted curve rather than a demonstrated property of the data, which are consistent with a log‐linear dose–response relationship.

### 3.4. Mediation Analysis

The drop in significance from Model 2 to Model 3 suggested that the albumin–DR association was largely mediated by renal microvascular damage rather than reflecting a direct link between albumin and the retina. To quantify that pathway, we ran a mediation analysis in the HbA1c ≥ 7.0% subgroup (*N* = 502; 223 DR events). Results are in Table 3. The indirect association (albumin ⟶ UACR ⟶ DR) was clearly different from zero (OR 0.79 per g/dL, 95% CI 0.61–0.92, bootstrap *p* < 0.001). The direct component was not significant (OR 0.63, 95% CI 0.30–1.26, bootstrap *p* = 0.179). The total effect was borderline (OR 0.50, *p* = 0.046), and about 34.4% of it was attributable to the UACR pathway. The confidence interval for the proportion mediated was wide (3.2%–198%), tracking the borderline total effect. We read this as supportive of the mediation hypothesis rather than as a precise point estimate, and replication is needed in larger longitudinal samples where the temporal ordering can be observed.

**TABLE 3:** Mediation analysis of the serum albumin–DR association through UACR (HbA1c ≥ 7.0%; *N* = 502).

Effect	Log‐OR (95% CI)	OR per 1 g/dL (95% CI)	*p* (bootstrap)
Natural indirect effect (via UACR)	−0.241 (−0.493, −0.080)	0.79 (0.61, 0.92)	< 0.001
Natural direct effect	−0.460 (−1.191, 0.229)	0.63 (0.30, 1.26)	0.179
Total effect	−0.702 (−1.489, −0.009)	0.50 (0.23, 0.99)	0.046
Proportion mediated, % (95% CI)	—	34.4% (3.2%, 198.1%)	—

*Note:* Mediator: log (UACR + 1). Both mediator and outcome models adjusted for age, sex, BMI, SBP, diabetes duration, and CRP. Bootstrap 95% CIs and two‐sided *p* values from 2000 nonparametric resamples; CIs are percentile‐based. The OR column is the exponentiated log‐OR column. Both bounds of the indirect effect lie below 1.0 because higher albumin is associated with lower UACR and therefore with lower odds of DR. The proportion mediated is a ratio whose denominator is the total effect. Its upper bound can exceed 100% when the total effect approaches zero, or when the direct and indirect components carry opposite signs. The interval reported here reflects the borderline total effect, not mediation of more than the whole association. Effect labels follow the conventional terminology of mediation analysis and do not imply that a causal ordering has been established in these cross‐sectional data.

### 3.5. Subgroup Analyses

**FIGURE 2:**
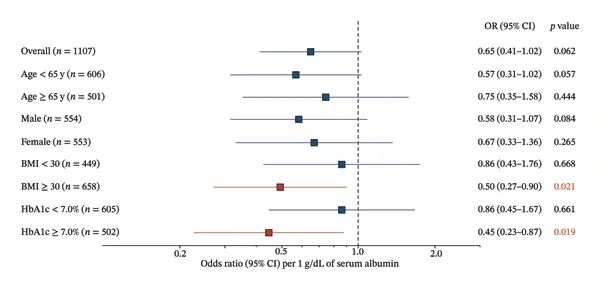
Forest plot of the association between serum albumin and diabetic retinopathy across prespecified subgroups. Odds ratios (ORs) are shown per 1‐g/dL increase in serum albumin from multivariable logistic regression in the full sample (overall, *n* = 1107) and in eight subgroups stratified by age (< 65 vs ≥ 65 years), sex, body‐mass index (< 30 vs ≥ 30 kg/m^2^), and glycemic control (HbA1c < 7.0% vs ≥ 7.0%). Each model retained the metabolic‐adjustment set (body‐mass index, systolic blood pressure, HbA1c, diabetes duration, and C‐reactive protein) plus whichever demographic covariate was not the stratifying variable. For example, age‐stratified models included sex, and sex‐stratified models included age. Squares mark the point estimate; horizontal lines span the 95% confidence interval. Red estimates have *p* < 0.05; navy estimates do not. The dashed vertical line at OR = 1.0 marks the null. The *x*‐axis is on a log scale. Numerical OR (95% CI) and *p* values are listed to the right of each row. The albumin × HbA1c interaction *p* value (not displayed in the plot) was 0.167, so the larger effect estimate in the HbA1c ≥ 7.0% stratum should be read as hypothesis‐generating rather than as a confirmed effect modification. OR, odds ratio; CI, confidence interval; BMI, body‐mass index; HbA1c, glycated hemoglobin.

Subgroup ORs are shown in Figure 2. Two strata stood out: patients with HbA1c ≥ 7.0% (OR 0.45 per 1 g/dL, 95% CI 0.23–0.87, *p* = 0.019) and obese patients with BMI ≥ 30 kg/m^2^ (OR 0.50 per 1 g/dL, 95% CI 0.27–0.90, *p* = 0.021). The corresponding reference strata showed no association. Both interaction *p* values exceeded 0.10. Multiplicative interaction tests carry little power at this sample size (Supporting Table S5), so a nonsignificant interaction is weak evidence of homogeneity. These subgroup results remain hypothesis‐generating and should not anchor stratified clinical decisions.

### 3.6. Predictive Performance

**FIGURE 3:**
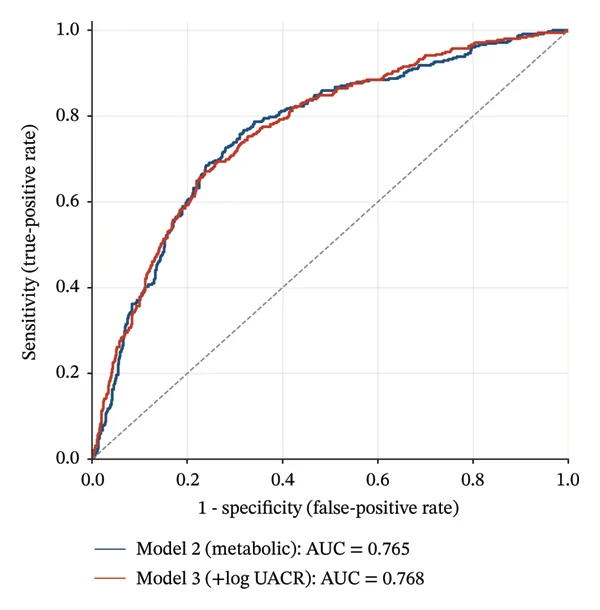
Receiver‐operating‐characteristic curves for two multivariable models forecasting diabetic retinopathy are shown for the full analytic sample (*N* = 1107; DR cases = 356). Model 2 (navy) adjusts for serum albumin, age, sex, body‐mass index, systolic blood pressure, HbA1c, diabetes duration, and C‐reactive protein (AUC = 0.765). Model 3 (red) adds log‐transformed urinary albumin‐to‐creatinine ratio to the same covariate set (AUC = 0.768). The diagonal gray reference line indicates the performance of a noninformative classifier. The negligible AUC gain from adding log UACR is consistent with the mediation result reported in Table 3—most of the apparent association between low serum albumin and DR runs through the renal pathway, leaving little independent predictive information to be added by UACR once albumin is already in the model. ROC, receiver‐operating characteristic; AUC, area under the curve; UACR, urinary albumin‐to‐creatinine ratio; DR, diabetic retinopathy.

Figure 3 compares the ROC curves for Models 2 and 3. The metabolic model alone produced an AUC of 0.765, and adding log‐UACR raised it only marginally to 0.768. Combined with the disappearance of the albumin coefficient after UACR adjustment, the small gain in discrimination is consistent with the idea that most of the prognostic value carried by albumin is already captured by UACR.

## 4. Discussion

In this NHANES sample of 1107 U.S. adults with diabetes, serum albumin tracked with DR in the simplest analyses but largely lost its independent footing once renal microvascular damage was taken into account. The pattern was sharpest in patients with poorly controlled diabetes. In that stratum, the metabolic‐adjusted association was strong but weakened to nonsignificance once UACR entered the model, and the mediation analysis attributed roughly a third of the total association to the renal pathway. The same sequence appeared in the survey‐weighted models, so it is not an artifact of ignoring the sampling design. NLR did not predict DR independently in any model. The most economical interpretation is that low albumin in diabetes is best read as a downstream signal of systemic microvascular leakage rather than as an upstream protective factor in its own right, which is the “renal–retinal axis” idea applied in a concrete, measurable way [23, 24]. This is an interpretation of associations measured at a single point in time, not evidence that albumin acts on the retina by way of the kidney.

This reading fits the common‐soil hypothesis for diabetic microvascular complications [25, 26]. Chronic hyperglycemia damages the endothelial glycocalyx and breaches the vascular barrier in multiple vascular beds simultaneously [27]. In the kidney, the breach appears as albuminuria and a fall in serum albumin; in the eye, it presents as a blood–retinal barrier breakdown and the lesions that define DR [28]. Read in that light, the antioxidant or oncotic roles of albumin are unlikely to be the dominant explanation for the albumin–DR association we observed. The biology that drives nephropathy is the same biology that drives retinopathy, and low albumin is one of its visible fingerprints.

Whether that logic extends across the severity spectrum is a separate question, and this study cannot answer it. Our outcome is binary, defined by an ETDRS severity score of 14 or above, so a single microaneurysm and active proliferative disease enter the analysis on the same footing despite being biologically distinct. Barrier permeability and capillary nonperfusion dominate early disease, which is what a systemic index of microvascular leakage might be expected to track. Advanced disease is a different problem. Fibrovascular proliferation, tractional distortion of the retinal layers, and photoreceptor loss are structural changes, driven largely by local transcriptional programs within the retina and vitreous [21]. They persist as measurable architectural damage after vitrectomy has removed the proliferative membranes [20]. There is no obvious mechanism by which a circulating marker of glomerular barrier failure would track that stage. Our results are therefore most plausibly read as showing that serum albumin, like UACR, indices the systemic permeability process that initiates and sustains early DR while carrying little information about local structural remodeling. Testing this would require severity‐graded outcomes. The NHANES grading supports that in principle, and it is the analysis we would prioritize in a longitudinal extension of this work.

Our null finding for NLR also fits a pattern noted in the literature for years [29, 30]. A 2025 meta‐analysis reported that NLR tends to run higher in DR patients but flagged substantial between‐study heterogeneity. That heterogeneity is itself the clue [31]. When we look at the cohorts reporting the strongest NLR–DR associations [32, 33], two features tend to co‐occur: the analyses adjust for age and sex but not for diabetes duration, HbA1c, blood pressure, and CRP simultaneously, and the populations are drawn from tertiary eye clinics where DR cases are tilted toward more advanced disease [34]. Both are conditions under which NLR, a nonspecific inflammatory marker that rises with chronic hyperglycemia, longer disease duration, and concurrent infection, can act as a proxy for unmeasured metabolic burden rather than as an independent predictor [35]. Once HbA1c, duration, and CRP enter the model, that proxy effect is absorbed and the residual NLR signal disappears, which is what we observed (Model 2 OR 1.06, *p* = 0.319; survey‐weighted OR 1.06, *p* = 0.478).

There is also a mechanistic reason not to expect much from a single leukocyte ratio. The inflammatory processes that matter in the diabetic retina are compartmentalized and begin early. Microglial activation, Müller‐cell gliosis, and ganglion‐cell loss are under way before the vascular lesions that define clinical DR become visible, and they are driven by local signaling rather than by circulating leukocyte traffic [8]. Mitochondrial dysfunction runs alongside this, and the retinal unfolded‐protein response it triggers has its own candidate biomarkers, none of which a complete blood count captures [15]. The markers that carry information about oxidative injury are similarly tissue‐derived, or depend on specialized assays rather than routine hematology [7]. Even at cohort scale, composite systemic inflammatory indices predict ocular disease only modestly [14]. Inflammation clearly matters in DR. The routine complete blood count is simply not a good instrument for measuring it. A single NLR value from a cross‐sectional blood draw cannot stratify DR risk in individual patients, even after standard metabolic covariates are accounted for. Repeated measurements, or panels combining inflammatory with neurodegenerative and oxidative markers, might do better, but our data cannot test that.

Clinically, the practical message is that low serum albumin in a patient with HbA1c ≥ 7.0% should not be reflexively read as a nutritional problem. It should trigger a parallel evaluation of the kidney and the retina during the same clinic visit. This is in line with the recent cross‐sectional report by Li et al. linking low albumin to higher DR risk in type 2 diabetes [36]. Treating albumin as a cheap, system‐level fingerprint of microvascular leakage gives the clinician an extra low‐cost trigger for the kind of dual screening that current guidelines already recommend in principle but seldom enforce in practice.

Dual screening has always been an operational problem: two clinics, two appointments, two sets of results. That is changing. Multitask deep‐learning frameworks pretrained on large retinal image collections can now screen for a range of systemic and metabolic conditions from a single fundus imaging session, returning several risk outputs at the point of care [37]. The workflow we are describing is therefore a matter of connecting tools that already exist. A low albumin value flags the patient. One fundus acquisition is performed at that visit, and the images are read for retinopathy and for renal and cardiometabolic risk at the same time. Our results bear on how such a system should be interpreted rather than on whether it can be built. If albumin and UACR largely report the same underlying permeability process, a screening pathway triggered by albumin will add most where UACR has not recently been measured and least where it has. This is worth checking before joint screening is promoted as routine.

### 4.1. Strengths and Limitations

This study draws on a large, nationally representative sample with centralized fundus grading at the Wisconsin Reading Center. Pairing restricted cubic splines for nonlinearity with a bootstrap‐based mediation analysis is uncommon in the NHANES biomarker‐and‐DR literature, and it provides a numerical estimate for a hypothesis that has so far rested mostly on mechanistic reasoning. The principal associations were reproduced under a design‐based, survey‐weighted analysis, which makes it unlikely that they are artifacts of the unweighted specification.

There are several caveats worth flagging openly. The cross‐sectional design cannot establish the temporal ordering that mediation analysis assumes; the model can only show that the data are compatible with the hypothesis, not that the causal arrows point that way. Low albumin, elevated UACR, and DR are all measured at the same instant, and a process in which albumin fell as a consequence of established nephropathy would produce the same decomposition. We have therefore used associational language throughout and reserve the causal reading for longitudinal work.

Second, and most consequential for the mediation estimate, we could not adjust for medications that act directly on the mediator. Renin–angiotensin–aldosterone system blockers, and in more recent cohorts SGLT2 inhibitors, reduce albuminuria substantially and are prescribed preferentially to patients who already have nephropathy or hypertension. The NHANES prescription files were not merged into our analytic dataset, so this treatment channel is entirely unmeasured. Two mechanisms then operate in opposite directions. Confounding by indication inflates the pathway. Patients with worse renal disease are more likely to be treated, so treatment status is positively associated with the underlying microvascular burden that drives both UACR and DR, and leaving it unmeasured inflates the apparent albumin–UACR–DR pathway. Pharmacological suppression of the mediator does the reverse. In treated patients, UACR sits below the level their underlying barrier damage would otherwise produce. That weakens the measured albumin ⟶ UACR path and degrades UACR as a marker of the DR‐relevant process, biasing the indirect effect toward the null and the proportion mediated downward. Which mechanism dominates depends on the treated fraction and on how closely treatment tracks severity in this sample, and neither can be estimated without the prescription data. The 34.4% figure should therefore be read as a point estimate from a model that does not adjust for treatment, and its already wide confidence interval understates the true uncertainty. NHANES releases the prescription files, so linking them is a feasible next step, and we would expect it to shift this estimate rather than confirm it.

Third, the analysis is constrained by precision as much as by sample size. The full sample could detect an albumin OR of 0.52 or more extreme at 80% power, and the poorly controlled subgroup an OR of 0.38. The multiplicative interaction test, which is the formally correct test of effect modification, had roughly 28% power against the difference we observed between strata. A nonsignificant interaction *p* value under those conditions is weak evidence of homogeneity, and the subgroup findings should be read in that light (Supporting Table S5).

Fourth, albumin, UACR, and NLR were each measured once, so they do not capture variability over time, and single‐occasion measurement will attenuate associations for all three. Fifth, the binary outcome lumps nonproliferative and proliferative DR together, and as discussed above the mediation pattern may differ across severity grades or in diabetic macular edema. Sixth, LDL‐C and triglycerides were available only in the fasting subsample, and the variables entering the models were handled by complete‐case selection, which excludes participants with missing covariates and could introduce selection bias if that missingness is informative. Finally, the fundus protocol used in NHANES 2005–2008 captured two fields per eye, so peripheral lesions outside those fields may be missed; the resulting misclassification would be expected to dilute the associations we report rather than create them.

## 5. Conclusions

In this nationally representative U.S. sample of adults with diabetes, serum albumin behaved as a downstream indicator of systemic microvascular leakage rather than as an independent protective factor for DR. The inverse association was confined to patients with HbA1c ≥ 7.0% and was largely mediated by renal microvascular damage as indexed by UACR, a pattern that persisted under survey‐weighted analysis. NLR added no independent predictive value. Because the data are cross‐sectional, these results describe an association structure and not a demonstrated causal sequence, and confirmation will require longitudinal cohorts with linked medication data. The practical implication is straightforward. When a clinician encounters low serum albumin in a patient with poor glycemic control, the kidneys and the retina warrant assessment at the same visit rather than in sequence. Emerging multitask retinal imaging systems make that increasingly practical within a single encounter.

## Author Contributions

Y.Z. and L.Y. conceived the study and designed the analytical plan. Y.Z., J.L., and L.Y. developed the method. Y.Z. and X.X. wrote the analysis code and produced the figures. J.L. and Q.Y. curated the data, and J.L., Q.Y., and X.X. validated the results. Y.Z. drafted the manuscript, and L.Y. revised it. L.Y. supervised the project and acted as guarantor.

## Funding

This research did not receive any specific grant from funding agencies in the public, commercial, or not‐for‐profit sectors.

## Disclosure

All authors read and approved the final version.

## Conflicts of Interest

The authors declare no conflicts of interest.

## Supporting Information

Additional supporting information can be found online in the Supporting Information section.

## Supporting information


**Supporting Information** Supporting Figure S1 (participant flow and analytical workflow), Supporting Table S1 (STROBE checklist), Supporting Table S2 (hypertension definition sensitivity analysis), Supporting Table S3 (missing‐data accounting), Supporting Table S4 (restricted cubic spline knot selection), Supporting Table S5 (statistical power and minimum detectable effects), Supporting Table S6 (mediation analysis specification), Supporting Table S7 (quartile‐based sensitivity analysis), and Supporting Table S8 (survey‐weighted sensitivity analysis) are provided as separate files.

## Data Availability

The NHANES data analyzed in this study are publicly available from the U.S. Centers for Disease Control and Prevention at https://www.cdc.gov/nchs/nhanes/index.html.
