# Supplementary material for: Serum Albumin as a Marker of Microvascular Burden in Diabetic Retinopathy: Mediation by Diabetic Nephropathy in Uncontrolled Diabetes
Source: J Ophthalmol. 2026 Sep 16;2026:6568455. doi: 10.1155/joph/6568455 (PMC13579243; doi:10.1155/joph/6568455)
Supplement: Supplementary file 1 [file JOPH-2026-6568455-s001.zip › Supplementary_Material_R1.docx]

**Supplementary Material**

Serum Albumin as a Marker of Microvascular Burden in Diabetic Retinopathy: Mediation by Diabetic Nephropathy in Uncontrolled Diabetes

*Revised version (R1) — Manuscript 6568455*

**This file contains:**

• Supplementary Table S1. STROBE checklist for cross-sectional studies.

• Supplementary Table S2. Sensitivity analysis: JNC7 versus 2017 ACC/AHA definition of hypertension.

• Supplementary Table S3. Missing-data accounting for all analysed variables.

• Supplementary Table S4. Restricted cubic spline knot selection by AIC and BIC.

• Supplementary Table S5. Statistical power and minimum detectable effects.

• Supplementary Table S6. Mediation analysis specification and equivalent code (R / Python).

• Supplementary Table S7. Sensitivity analysis: odds ratios for DR by quartiles of serum albumin.

• Supplementary Table S8. Survey-weighted sensitivity analysis of Models 1–3.

• Supplementary Figure S1. Participant selection and analytical workflow (provided as a separate image file: FigS1_Flowchart.png / .pdf).

*Tables S2–S5 and S8 are new in this revision and were added in response to the reviewer's comments. Tables previously numbered S2 (quartile sensitivity) and S3 (mediation specification) are now S7 and S6 respectively, so that all supplementary tables are numbered in the order in which they are first cited in the manuscript.*

**Supplementary Table S1.** STROBE statement — checklist of items to include in reports of cross-sectional studies

*Item-by-item indication of where each STROBE element is addressed in the revised manuscript (M = Methods section §; R = Results section §; D = Discussion; A = Abstract; T = Table; F = Figure).*

| **Item No.** | **Recommendation** | **Specification** | **Section in manuscript** |
| --- | --- | --- | --- |
| 1 | Title and abstract | (a) Indicate the study's design with a commonly used term in the title or the abstract | Title; Abstract (Methods) |
| 1 |  | (b) Provide in the abstract an informative and balanced summary of what was done and what was found | Abstract |
| 2 | Background/rationale | Explain the scientific background and rationale for the investigation being reported | Introduction §1, ¶1–4 |
| 3 | Objectives | State specific objectives, including any pre-specified hypotheses | Introduction §1, last ¶ |
| 4 | Study design | Present key elements of study design early in the paper | Methods §2.1 |
| 5 | Setting | Describe the setting, locations, and relevant dates | Methods §2.1 |
| 6 | Participants | (a) Eligibility criteria, sources and methods of selection of participants | Methods §2.2; Supp Fig S1 |
| 7 | Variables | Clearly define all outcomes, exposures, predictors, potential confounders, and effect modifiers | Methods §2.3, §2.4 |
| 8 | Data sources/measurement | For each variable of interest, give sources of data and details of methods of assessment (measurement). Describe comparability of assessment methods if there is more than one group | Methods §2.3, §2.4 |
| 9 | Bias | Describe any efforts to address potential sources of bias | Methods §2.5; Discussion §4.1 (Limitations) |
| 10 | Study size | Explain how the study size was arrived at | Methods §2.2; Supp Fig S1; Supp Table S5 |
| 11 | Quantitative variables | Explain how quantitative variables were handled in the analyses. If applicable, describe which groupings were chosen and why | Methods §2.4, §2.5; Supp Tables S4, S7 |
| 12 | Statistical methods | (a) Describe all statistical methods, including those used to control for confounding | Methods §2.5 |
| 12 |  | (b) Describe any methods used to examine subgroups and interactions | Methods §2.5; Supp Table S5 |
| 12 |  | (c) Explain how missing data were addressed | Methods §2.4; Supp Table S3 |
| 12 |  | (d) If applicable, describe analytical methods taking account of sampling strategy | Methods §2.5; Supp Table S8 |
| 12 |  | (e) Describe any sensitivity analyses | Methods §2.5; Supp Tables S2, S4, S7, S8 |
| 13 | Participants | (a) Report the numbers of individuals at each stage of the study | Results §3.1; Supp Fig S1 |
| 13 |  | (b) Give reasons for non-participation at each stage | Supp Fig S1 |
| 13 |  | (c) Consider use of a flow diagram | Supp Fig S1 |
| 14 | Descriptive data | (a) Give characteristics of study participants (demographic, clinical, social) | Results §3.1; Table 1 |
| 14 |  | (b) Indicate the number of participants with missing data for each variable of interest | Methods §2.4; Supp Table S3 |
| 15 | Outcome data | Report numbers of outcome events or summary measures | Results §3.1; Table 1 |
| 16 | Main results | (a) Give unadjusted estimates and, if applicable, confounder-adjusted estimates and their precision (e.g. 95% CI). Make clear which confounders were adjusted for and why they were included | Results §3.2; Table 2; Supp Table S8 |
| 16 |  | (b) Report category boundaries when continuous variables were categorized | Supp Table S7 |
| 16 |  | (c) If relevant, consider translating estimates of relative risk into absolute risk for a meaningful time period | Results §3.3; Figure 1 |
| 17 | Other analyses | Report other analyses done — eg analyses of subgroups and interactions, and sensitivity analyses | Results §3.2–§3.6; Figures 1–3; Table 3; Supp Tables S2, S4, S5, S7, S8 |
| 18 | Key results | Summarise key results with reference to study objectives | Discussion §4, ¶1 |
| 19 | Limitations | Discuss limitations of the study, taking into account sources of potential bias or imprecision. Discuss both direction and magnitude of any potential bias | Discussion §4.1 (Limitations) |
| 20 | Interpretation | Give a cautious overall interpretation of results considering objectives, limitations, multiplicity of analyses, results from similar studies, and other relevant evidence | Discussion §4 |
| 21 | Generalisability | Discuss the generalisability (external validity) of the study results | Discussion §4.1; Supp Table S8 |
| 22 | Funding | Give the source of funding and the role of the funders for the present study and, if applicable, for the original study on which the present article is based | Funding statement |

*STROBE = Strengthening the Reporting of Observational Studies in Epidemiology. The checklist is available at http://www.strobe-statement.org/.*

**Supplementary Table S2.** Sensitivity analysis: JNC7 versus 2017 ACC/AHA definition of hypertension

*Hypertension is used in the manuscript only as a descriptive variable in Table 1 and as a stratifying variable in the Figure 2 subgroup analysis. The adjustment set of Models 2 and 3 contains systolic blood pressure as a continuous covariate, so the choice of threshold cannot in itself alter the primary estimates. The models below verify this directly by adding each binary definition to the published models.*

| **Definition** | **Threshold** | **n classified as hypertensive** | **Prevalence** |
| --- | --- | --- | --- |
| JNC7 (as used in Table 1) | SBP ≥ 140 or DBP ≥ 90 mmHg | 388 | 35.0% |
| 2017 ACC/AHA | SBP ≥ 130 or DBP ≥ 80 mmHg | 708 | 64.0% |

| **Population** | **Model** | **Albumin OR (95% CI)** | **P** |
| --- | --- | --- | --- |
| Full sample (N = 1,107) | Model 2, as published (SBP continuous) | 0.65 (0.41–1.02) | 0.062 |
|  | Model 2 + hypertension (JNC7) | 0.64 (0.41–1.02) | 0.061 |
|  | Model 2 + hypertension (ACC/AHA) | 0.64 (0.41–1.02) | 0.061 |
|  | Model 3, as published | 0.82 (0.51–1.31) | 0.399 |
|  | Model 3 + hypertension (ACC/AHA) | 0.81 (0.51–1.31) | 0.395 |
| HbA1c ≥ 7.0% (N = 502) | Model 2, as published | 0.45 (0.23–0.87) | 0.019 |
|  | Model 2 + hypertension (JNC7) | 0.45 (0.23–0.88) | 0.019 |
|  | Model 2 + hypertension (ACC/AHA) | 0.44 (0.23–0.87) | 0.018 |
|  | Model 3, as published | 0.56 (0.28–1.13) | 0.107 |
|  | Model 3 + hypertension (ACC/AHA) | 0.56 (0.28–1.13) | 0.106 |

| **Definition** | **Stratum** | **n** | **Albumin OR (95% CI)** | **P** | **P for interaction** |
| --- | --- | --- | --- | --- | --- |
| JNC7 | Hypertensive | 388 | 0.83 (0.39–1.77) | 0.631 | 0.754 |
|  | Normotensive | 719 | 0.56 (0.31–1.01) | 0.053 |  |
| ACC/AHA | Hypertensive | 708 | 0.57 (0.33–1.01) | 0.053 | 0.298 |
|  | Normotensive | 399 | 0.93 (0.41–2.11) | 0.855 |  |

*Odds ratios are per 1 g/dL increase in serum albumin. Diastolic blood pressure was taken as the mean of the available readings (BPXDI1–BPXDI4), with zero values treated as missing. Adopting the lower ACC/AHA threshold nearly doubles the proportion classified as hypertensive but leaves every albumin estimate unchanged to within 0.01 on the OR scale. Neither definition produces a significant albumin × hypertension interaction.*

**Supplementary Table S3.** Missing-data accounting for all analysed variables

*(a) Variables entering Models 1–3, the spline models and the mediation analysis. The analytic sample was assembled by complete-case selection on these variables, so none of them is missing in the final sample of 1,107 participants; participants with any missing value were excluded at the sample-selection stage shown in Supplementary Figure S1. No imputation was used for any modelled variable.*

| **Variable** | **Role in the analysis** | **Observed** | **Missing** | **Handling** |
| --- | --- | --- | --- | --- |
| Serum albumin | Exposure | 1,107 | 0 (0.0%) | complete case |
| UACR | Mediator / covariate in Model 3 | 1,107 | 0 (0.0%) | complete case |
| NLR | Secondary exposure | 1,107 | 0 (0.0%) | complete case |
| Diabetic retinopathy | Outcome | 1,107 | 0 (0.0%) | complete case |
| Age | Covariate | 1,107 | 0 (0.0%) | complete case |
| Sex | Covariate | 1,107 | 0 (0.0%) | complete case |
| Body-mass index | Covariate | 1,107 | 0 (0.0%) | complete case |
| Systolic blood pressure | Covariate | 1,107 | 0 (0.0%) | complete case |
| HbA1c | Covariate / stratifying variable | 1,107 | 0 (0.0%) | complete case |
| Diabetes duration | Covariate | 1,107 | 0 (0.0%) | complete case |
| C-reactive protein | Covariate | 1,107 | 0 (0.0%) | complete case |

*(b) Descriptive variables that appear only in Table 1. These were merged into the dataset after the analytic sample had been fixed and therefore did contain missing values. Median imputation was applied to waist circumference and diastolic blood pressure for the purposes of Table 1 only; neither variable enters any regression model. The fasting-subsample lipids are reported as observed, without imputation.*

| **Variable** | **Observed** | **Missing** | **% missing** | **Handling** |
| --- | --- | --- | --- | --- |
| Waist circumference | 1,066 | 41 | 3.7% | median imputation (Table 1 only) |
| Diastolic blood pressure | 1,024 | 83 | 7.5% | median imputation (Table 1 only) |
| HDL-C | 1,061 | 46 | 4.2% | reported as observed |
| LDL-C | 494 | 613 | 55.4% | fasting subsample; reported as observed |
| Triglycerides | 529 | 578 | 52.2% | fasting subsample; reported as observed |

*Percentages are of the 1,107 participants in the analytic sample. LDL-C and triglycerides were measured only in the morning fasting subsample of NHANES, which accounts for their high missingness; the observed sample sizes are also given in the footnote to Table 1. Because the modelled variables were handled by complete-case selection, informative missingness could in principle bias the analytic sample. This is addressed in the Limitations rather than by an imputation model.*

**Supplementary Table S4.** Restricted cubic spline knot selection by AIC and BIC

*Models were fitted in the HbA1c ≥ 7.0% subgroup (N = 502, DR = 223), adjusted for age, sex, body-mass index, systolic blood pressure, diabetes duration and C-reactive protein — the specification shown in Figure 1. Knots were placed at the percentiles recommended by Harrell for each knot count. The test of non-linearity is a likelihood-ratio test of the spline terms beyond the linear term, against the corresponding linear model.*

| **Model** | **Knot percentiles** | **Knot values (g/dL)** | **df** | **AIC** | **BIC** | **P for non-linearity** |
| --- | --- | --- | --- | --- | --- | --- |
| Linear (no spline) | — | — | 7 | 610.57 | 644.32 | — (reference) |
| RCS, 3 knots | 10, 50, 90 | 3.7, 4.1, 4.5 | 8 | 612.38 | 650.35 | 0.664 |
| RCS, 4 knots | 5, 35, 65, 95 | 3.5, 4.0, 4.2, 4.6 | 9 | 613.88 | 656.06 | 0.705 |
| RCS, 5 knots | 5, 27.5, 50, 72.5, 95 | 3.5, 3.9, 4.1, 4.2, 4.6 | 10 | 613.48 | 659.89 | 0.378 |

*AIC = Akaike information criterion; BIC = Bayesian information criterion; RCS = restricted cubic spline; df = model degrees of freedom. Among the spline specifications, three knots gave the lowest value of both criteria, and this is the model presented in Figure 1. Two things temper that choice. The differences between the three spline models are small (ΔAIC < 1.6), so the ranking is not strong evidence against the four- and five-knot alternatives. More importantly, none of the spline models improved on the simple linear model by AIC or BIC, and the likelihood-ratio test of non-linearity was not significant at any knot count. The dose–response relationship is therefore statistically consistent with log-linearity, and the curvature visible in Figure 1 is described in the text as a feature of the fitted curve rather than as an established departure from linearity.*

**Supplementary Table S5.** Statistical power and minimum detectable effects

*Minimum detectable effects were computed from the observed standard error of the serum albumin log-odds coefficient in each fitted model, using |log OR| ≥ (z₀.₉₇₅ + z₀.₈₀) × SE = 2.802 × SE, i.e. the effect size that would be detected with 80% power at a two-sided α of 0.05. Achieved power is the power of the same test against the effect actually observed.*

| **Analysis** | **n** | **DR events** | **SE of log OR** | **Observed OR** | **Minimum detectable OR** | **Achieved power** |
| --- | --- | --- | --- | --- | --- | --- |
| Full sample, Model 2 | 1,107 | 356 | 0.234 | 0.65 | 0.52 | 46% |
| Full sample, Model 3 | 1,107 | 356 | 0.241 | 0.82 | 0.51 | 13% |
| HbA1c < 7.0%, Model 2 | 605 | 133 | 0.336 | 0.86 | 0.39 | 7% |
| HbA1c ≥ 7.0%, Model 2 | 502 | 223 | 0.344 | 0.45 | 0.38 | 65% |
| HbA1c ≥ 7.0%, Model 3 | 502 | 223 | 0.358 | 0.56 | 0.37 | 36% |

| **Quantity** | **Value** |
| --- | --- |
| Interaction coefficient (albumin × HbA1c ≥ 7.0%), log-OR | −0.612 |
| Standard error | 0.443 |
| P value | 0.167 |
| Ratio of odds ratios (HbA1c ≥ 7.0% vs < 7.0%) | 0.54 (95% CI 0.23–1.29) |
| Ratio of odds ratios detectable at 80% power | ≤ 0.29 (or ≥ 3.46) |
| Achieved power of the interaction test | 28% |
| DR events, HbA1c < 7.0% | 133 of 605 (22.0%) |
| DR events, HbA1c ≥ 7.0% | 223 of 502 (44.4%) |
| Events per variable, HbA1c ≥ 7.0% Model 3 | 27.9 |

*Two statements in the manuscript rest on this table. The first is that the smaller HbA1c ≥ 7.0% subgroup reached significance where the larger full sample did not because of effect size, not chance. The subgroup estimate (OR 0.45) is close to that subgroup's minimum detectable effect (0.38), while the full-sample estimate (OR 0.65) falls well short of its own (0.52). DR events are also concentrated in the poorly controlled stratum (223 of 356). The second is that the non-significant interaction term is not evidence of homogeneity. That test had roughly 28% power against the observed between-stratum difference, and would have needed a ratio of odds ratios about twice as extreme as the one observed to reach significance. Power calculated from observed estimates is descriptive. We report it to characterise the precision of the design, not to justify any result after the fact.*

**Supplementary Table S6.** Specification of the mediation analysis

| **Item** | **Specification** |
| --- | --- |
| Exposure (X) | Serum albumin (continuous, per 1 g/dL) |
| Mediator (M) | log(UACR + 1), with UACR in mg/g; +1 added before log to handle zero values |
| Outcome (Y) | Diabetic retinopathy (binary; ETDRS severity ≥ 14 in either eye) |
| Confounders (C) | Age, sex, body mass index, systolic blood pressure, diabetes duration, C-reactive protein. Same set adjusted for in both mediator and outcome models. |
| Sub-population | Participants with HbA1c ≥ 7.0% (N = 502, DR events = 223) |
| Mediator model | Linear regression: log(UACR+1) = α₀ + a·ALBUMIN + γᵀC + ε |
| Outcome model | Logistic regression: logit P(Y=1) = β₀ + c'·ALBUMIN + b·log(UACR+1) + δᵀC |
| Indirect effect (NIE) | a × b on the log-odds scale (product-of-coefficients, rare-outcome approximation) |
| Direct effect (NDE) | c' on the log-odds scale |
| Total effect | c' + a × b on the log-odds scale |
| Proportion mediated | (a × b) / (c' + a × b) |
| Inference | Non-parametric bootstrap with 2,000 resamples; percentile-based 95% CIs and two-sided P values |
| Random seed | 20260527 (for reproducibility) |
| Software | Python 3.9 — pandas 2.x, statsmodels 0.14, NumPy 1.26 |
| Equivalent in R | mediation::mediate(model.m, model.y, treat='ALBUMIN', mediator='logUACR', boot=TRUE, sims=2000) |
| Identification assumptions | No unmeasured exposure–outcome, mediator–outcome or exposure–mediator confounding, and a temporal ordering X → M → Y. The last of these cannot be verified in a cross-sectional design, and unmeasured mediator–outcome confounding by RAAS blockade and SGLT2 inhibition is discussed in the Limitations. |

*Note. The proportion mediated is a ratio of two coefficient products and therefore carries a wider confidence interval than either component, particularly when the denominator (the total effect) lies close to zero. Its upper bound can exceed 100% when the total effect approaches zero or when the direct and indirect components carry opposite signs; an interval whose upper limit exceeds 100% should therefore be read as an indication of imprecision rather than as mediation of more than the entire association. A point estimate of 34.4% (bootstrap 95% CI 3.2%–198.1%) was obtained in the present sub-sample. The result is reported as supportive rather than conclusive, and longitudinal validation is encouraged.*

**Supplementary Table S7.** Sensitivity analysis: odds ratios for DR by quartile of serum albumin

*Multivariable logistic regression with Q1 (lowest albumin) as reference. All models adjusted for age, sex, BMI, systolic blood pressure, HbA1c, diabetes duration and C-reactive protein. The HbA1c subgroup model omits HbA1c, because it is the stratifying variable.*

| **Population** | **Quartile** | **Albumin range (g/dL)** | **OR (95% CI)** | **P value** |
| --- | --- | --- | --- | --- |
| Full sample (N = 1,107) | Q1 (reference) | ≤ 3.9 | 1.00 (ref) | — |
|  | Q2 | 3.9 – 4.1 | 0.83 (0.57–1.19) | 0.309 |
|  | Q3 | 4.1 – 4.3 | 0.76 (0.51–1.14) | 0.190 |
|  | Q4 | > 4.3 | 0.78 (0.51–1.20) | 0.267 |
|  | P for trend |  |  | 0.221 |
| HbA1c ≥ 7.0% (N = 502) | Q1 (reference) | ≤ 3.9 | 1.00 (ref) | — |
|  | Q2 | 3.9 – 4.1 | 0.69 (0.41–1.15) | 0.151 |
|  | Q3 | 4.1 – 4.3 | 0.80 (0.46–1.40) | 0.444 |
|  | Q4 | > 4.3 | 0.77 (0.40–1.45) | 0.414 |
|  | P for trend |  |  | 0.425 |

*Quartile cut-points were derived from the full analytic sample. P for trend was obtained by entering the quartile rank (1–4) as a continuous variable in the same multivariable model. Categorising a continuous exposure into quartiles discards within-quartile variation and reduces power, which is the most likely explanation for the attenuated and non-significant quartile estimates relative to the continuous analysis in Table 2.*

**Supplementary Table S8.** Survey-weighted sensitivity analysis of Models 1–3

*Design-based re-analysis of the primary models. Weights are the four-year mobile examination centre weight for the combined 2005–2006 and 2007–2008 cycles (WTMEC2YR / 2), with SDMVSTRA as the stratum variable and SDMVPSU as the primary sampling unit. Variances were obtained by Taylor-series linearization. The HbA1c strata were analysed as design domains, so that non-domain observations contribute to the variance through the full design rather than being deleted. The analytic sample spans 31 strata and 62 primary sampling units, with no single-PSU stratum; degrees of freedom for the t reference distribution are 31.*

| **Population** | **Model** | **Unweighted OR (95% CI)** | **P** | **Weighted OR (95% CI)** | **P** |
| --- | --- | --- | --- | --- | --- |
| Full sample (N = 1,107) | Model 1 (unadjusted) | 0.61 (0.41–0.89) | 0.011 | 0.54 (0.34–0.85) | 0.010 |
|  | Model 2 (metabolic) | 0.65 (0.41–1.02) | 0.062 | 0.59 (0.35–1.00) | 0.050 |
|  | Model 3 (+ log UACR) | 0.82 (0.51–1.31) | 0.399 | 0.67 (0.38–1.19) | 0.162 |
| HbA1c < 7.0% (n = 605) | Model 1 (unadjusted) | 0.93 (0.50–1.70) | 0.807 | 0.82 (0.43–1.56) | 0.530 |
|  | Model 2 (metabolic) | 0.86 (0.45–1.67) | 0.661 | 0.82 (0.34–1.98) | 0.655 |
|  | Model 3 (+ log UACR) | 1.08 (0.55–2.10) | 0.822 | 0.97 (0.39–2.46) | 0.955 |
| HbA1c ≥ 7.0% (n = 502) | Model 1 (unadjusted) | 0.61 (0.36–1.04) | 0.068 | 0.54 (0.28–1.04) | 0.063 |
|  | Model 2 (metabolic) | 0.45 (0.23–0.87) | 0.019 | 0.41 (0.17–0.99) | 0.047 |
|  | Model 3 (+ log UACR) | 0.56 (0.28–1.13) | 0.107 | 0.44 (0.18–1.10) | 0.078 |
| NLR, full sample | Model 2 (metabolic) | 1.06 (0.94–1.20) | 0.319 | 1.06 (0.90–1.23) | 0.478 |
|  | Model 3 (+ log UACR) | 1.01 (0.89–1.15) | 0.885 | 1.03 (0.87–1.22) | 0.729 |

| **Quantity** | **Unweighted** | **Survey-weighted** |
| --- | --- | --- |
| DR prevalence | 32.2% (356 / 1,107) | 29.0% (population-projected) |

*Odds ratios are per 1 g/dL increase in serum albumin, except for the NLR rows, which are per one unit of NLR. The weighted analysis reproduces every feature of the unweighted analysis on which the manuscript's conclusions rest: an inverse association in the unadjusted model, attenuation on metabolic adjustment, further attenuation and loss of significance once log UACR is entered, the concentration of the signal in the HbA1c ≥ 7.0% stratum, and the null result for NLR. The weighted point estimates are slightly further from the null and their confidence intervals slightly wider, as expected when the design effect is taken into account. The two sets of estimates answer different questions. The weighted models describe the U.S. adult diabetic population; the unweighted models describe the association within the analysed sample. The primary models remain unweighted because the mediation estimator is not implemented for survey-weighted designs, and a partially weighted analysis would not be internally consistent.*

*End of Supplementary Material.*
